# The Impact of COVID-19 on Cancer Recurrence: A Narrative Review

**DOI:** 10.34172/aim.2022.74

**Published:** 2022-07-01

**Authors:** Ahmad R Mafi, Ali Ghanbari Motlagh, Payam Azadeh

**Affiliations:** ^1^Radiation Oncology Department, Imam Hossein Hospital, School of Medicine, Shahid Beheshti University of Medical Sciences, Tehran, Iran

**Keywords:** Angiotensin converting enzyme, Cancer, COVID-19, Dormant cancer cells, Inflammation, Interleukins, Metastasis, Neutrophil extracellular traps, Recurrence

## Abstract

Severe acute respiratory syndrome coronavirus 2 (SARSCoV-2) continues to be a worldwide healthcare problem. While our knowledge of the interaction of cancer and its management with COVID-19 mortality is gradually evolving, there are still many unanswered questions regarding the impact of COVID-19 on cancer and its prognosis. Several factors activated during COVID-19 have been implicated in tumorigenesis and the development of metastasis. Inflammation, hypoxia, reduced levels of angiotensin converting enzyme 2, elevated levels of Interleukin 6 and some other cytokines that are hallmarks of COVID-19 are capable of inducing tumor relapse and metastasis. On the other hand, there are reports that COVID-19 has been associated with cancer cure. Understanding the interaction between COVID-19 and tumor cells is essential for evaluating the potential long-term risks of COVID-19 in cancer patients, and for scheduling necessary preventive and therapeutic interventions. In this review, we briefly overview the potential impacts that COVID-19 might have on tumorigenesis and cancer relapse, as well as the role that COVID-19 might play in cancer remission and cure.

## Introduction

 Severe acute respiratory syndrome coronavirus 2 (SARSCoV-2) continues to be a worldwide healthcare problem. Since the beginning of the coronavirus disease (COVID-19) pandemic, several studies have reported higher COVID-19-related mortality in patients with cancer.^1^ While our knowledge of the interaction of cancer and its management with COVID-19 mortality is gradually evolving, there are still many unanswered questions regarding the impact of COVID-19 on cancer and its prognosis.^1^

 Recently, concerns have been raised regarding the main events that occur during severe COVID-19. Some of these events such as immune-mediated tissue inflammation and cytokine release, impairment of T-cell and natural killer (NK) cell activity and neutrophil hyperactivation may cause dormant cancer cells (DCCs) to reactivate – a phenomenon which might result in tumor recurrence and metastasis.^1,2^ Several proteins involved in cancer pathogenesis can be recruited by SARS-CoV-2. This event can alter cellular replication, metabolism, and epigenetic regulation leading to DNA damage and tumorigenesis.^1,2^

 In order to assess and predict the potential long-term risks of COVID-19 in cancer patients and to schedule proper preventive and therapeutic interventions, it is essential to understand the effects of COVID-19 on tumor cells and their microenvironment.^1^

 The impact of COVID-19 on cancer mortality is twofold: the first impact is attributed to infrastructure limitations (staff shortage, lock-downs, and deprioritization of elective clinical or non-emergency services), which is out of the scope of this paper.

 In this narrative review, we briefly overview the second impact, which is the potential effects that COVID-19 might have on tumorigenesis and cancer relapse, as well as the interesting and unexpected role that COVID-19 might play in cancer remission and cure.

## Viruses and Cancer

 Viral infections have been a great challenge for patients with cancer and oncologists for the past several decades. While some oncolytic viruses such as Coxsackie virus, vaccinia virus and adenovirus can cause tumor regression in various cancer types, some other oncogenic viruses such as HBV, HCV, HPV, EBV and HIV are known to participate in oncogenesis.^2^ Both viruses and cancer cells can express proteins that are recognizable by host T cells, resulting in T cell-mediated inflammation.^2^

 The chronic inflammation caused by viral infections has been recognized to be associated with increased tumor growth and metastasis.^2^ What links the pathogenesis of cancer and viral infections is that the immune system loses its ability to distinguish between self and non-self.

 Since the beginning of the SARS-CoV-2 pandemic, several studies have suggested that patients with cancer were more susceptible to developing severe infection and had a higher rate of mortality from COVID-19.^3,4^ Emerging evidence has shown a higher mortality rate in patients with COVID-19 and cancer, especially in those who suffered from progressive disease, hematological malignancies and recent antineoplastic therapies. This higher mortality rate, to some extent, could be explained by the fact that COVID-19 is accompanied by uncontrolled immune cell activation.^1,3-5^

 Besides, although there is no clear evidence that COVID-19 might cause or modulate cancer pathobiology, there are suggestions from experts that patients with a history of cancer who develop COVID-19 are probably at higher risk of “cancer-related death”. This higher risk could be due to immunosuppression, increased rate of co-morbidities, and in the case of lung cancer, underlying pulmonary compromise.^2,3^ Several mechanisms that are activated during coronavirus infection have been previously shown to have impacts on tumorigenesis and the development of metastasis. Therefore, it seems prudent to assume that COVID-19 may be able to create a microenvironment favorable to cancer recurrence.

 While our level of understanding of the impact of cancer and anticancer therapies on COVID-19 mortality is increasing, there is still little known about the possible mechanisms by which SARS-CoV-2 infection might interact with cancer cells. Therefore, potential long-term effects of COVID-19 on cancer outcome remains elusive.^1,2^

 Understanding the mechanism of interference of COVID-19 with tumor cells and their microenvironment seems to be essential for a precise evaluation of the potential long-term impacts of COVID-19 in the outcomes of cancer patients.^1^

 Recent data suggest that the inflammation induced by COVID-19 may affect tumor cells and their microenvironment, including DCCs.^6^ DCCs are rare and elusive cells that maintain and survive after apparently successful treatment. They usually disseminate early and localize in specific compartments of metastasis-prone distant organs, where they enter a clinically asymptomatic period called metastatic dormancy. Although tumoral cells do not proliferate in this period, they retain their proliferation ability, which can be activated under the influence of several intrinsic and extrinsic stimulants.^6^

 At this period, DCCs are rarely detectable with current diagnostic systems. At some point, DCCs might become reactivated in response to microenvironmental triggers such as inflammatory or immune-mediated signals and cause disease relapse and metastasis after successful treatment of the primary tumor. It has been suggested that all patients with a previous history of cancer may have DCCs.^6-8^

 Severe COVID-19 is accompanied by immune-mediated adverse events including tissue inflammation, impairment of the activity of T-cell and NK cells, neutrophil hyper-activation and thrombocytosis. It also interferes with cellular replication, metabolism and other regulatory mechanisms that are involved in tumorigenesis.^6^

## Inflammation

 More than 90% of cancer-related deaths occur due to metastatic recurrence, which in the majority of cases happens after the seemingly successful treatment of the cancer. Several factors are involved in the occurrence of metastasis, including the ability of tumor cells to migrate to other organs and proliferate, in either the primary or metastatic sites. This proliferation often happens following an asymptomatic period called metastatic dormancy.^1^

 This dormancy allows DCCs to escape from immune surveillance and therefore, to persist for a long time at distant sites, and to recur from a few months to several years after the treatment.^1,8^ The exact mechanisms involved in DCCs reactivation are only partly understood.

 Previous studies have shown that intrinsic tumor cell signals (such as mutations) may be responsible for this event. Besides, recent studies suggest that metastatic recurrence might be also related to microenvironmental factors such as inflammatory or immune-mediated signals.^1,9^

 Acute or chronic inflammation can trigger metastatic reawakening by disruption of tissue homeostasis.^1,9^

 Many inflammatory events such as infection, obesity, or even surgical removal of the primary tumor have been reported to enhance the migration of tumoral cells to metastatic sites and the reactivation of DCCs.^1^

 Apart from reawakening the DCCs, acute infection by respiratory viruses might result in CD8 + T-cells exhaustion, which may help premetastatic cells escape from immune-mediated control.^1^ Altogether, the above-mentioned evidence indicates that inflammatory events such as pathogen-induced inflammation, probably play an important role in inducing DCCs reawakening and the development of cancer recurrence or metastasis.^1^

## Hypoxia

 Pathological hypoxia is a common microenvironment factor in tumors that helps tumoral cell survive. This is partly due to neovascularization and enhanced angiogenesis that facilitates extravasation and dissemination of tumoral cells to other regions of the body which enhances invasiveness and risk of metastasis.^10,11^ COVID-19 is frequently accompanied by thrombosis and tissue hypoxia, which is a microenvironmental hallmark of poor prognosis in solid tumors.

 One rather unique characteristic of COVID-19 is that some patients may have significantly reduced oxygen saturations (measured by a pulse oximeter) without apparent signs of respiratory distress. This entity is called silent hypoxia. Recent studies have reported that between 20% and more than 40% of patients with COVID-19 might suffer from silent hypoxia.^12^

 Hypoxic microenvironments caused by COVID-19 might have a dual effect on DCCs: on the one hand, it can promote dormancy, and on the other, it can generate a drug-resistant aggressive phenotype that is capable of inducing tumor relapse and metastasis.^6^

## Angiotensin Converting Enzyme 2

 Renin-angiotensin system of both host and tumor cells plays a key role in tumor progression and angiogenesis in some types of cancers such as lung cancer.^13,14^ Shortly after the beginning of the COVID-19 pandemic, it was shown that angiotensin converting enzyme 2 (ACE2) was the cell surface binding site for the coronavirus SARS-CoV-2 entry and disease development.^1^ Recent studies have proposed that an imbalance in the action of ACE1- and ACE2-derived peptides, can enhance angiotensin II signaling, which is considered a primary driver of COVID-19 pathophysiology.^15^

 One of the trigger factors involved in the development of severe COVID-19 consequences seems to be ACE2 downregulation caused by viral infection.^1^

 During cell entry, SARS-CoV-2 binds to ACE2, which results in a reduction in the levels of functional ACE2. Decreased levels and inhibition of ACE2 limit the conversion of angiotensin II to angiotensin 1-7, which is known to have antiproliferative and anti-inflammatory effects.^16^ Previous reports have indicated that ACE2 can exert several anti-tumor effects including suppression of cancer angiogenesis and metastasis. As a result, ACE2 downregulation may be a contributing factor to tumor progression and metastasis development.^1,14,17^

 Compared to healthy individuals, expression levels of ACE2 are different in cells and tissues of patients with cancer, and the expression increases with malignancy grade, being higher in adenocarcinoma than adenoma of the colon, and higher in adenoma than normal colonic tissue. In addition, elevated levels of angiotensin II may increase angiotensin III levels, which further promotes cancer development and progression by enhancing tumorigenesis and angiogenesis.^2,16^

 The influence of angiotensin II/ angiotensin II type 1 receptor (AngII/AT1R) axis on a variety of non-immune cells can activate nuclear factor kB (NF-kB). NF-kB is a transcription factor involved in inflammatory responses.^1^

 It is one of the predominant molecules which links inflammation to cancer. NF-kB activation in tumoral cells enhances proliferation, chemoresistance, and invasion. In addition, it stimulates angiogenesis and immune suppression in the tumor microenvironment (TME), collectively promoting the metastatic process.^1^

 It must be mentioned, however, that ACE2 might play a dual role in carcinogenesis. There are reports arguing that ACE2 might be able to prevent the progression of hepatocellular carcinoma and non-small cell lung cancer and breast by angiogenesis inhibition.^17^ Further research is needed to clarify the exact nature of these complex interactions.

## Interleukin 6, Interleukin 1 and Other Cytokines

 Cytokine release syndrome is a life-threatening consequence of severe COVID-19.^1^ Reports have shown that many cytokines might be elevated in the plasma of patients with severe COVID-19, including those that can affect the cancer progression. These cytokines include (but are not limited to) tumor necrosis factor-α (TNF-α), vascular endothelial growth factor (VEGF), interferon γ (IFN-γ), interleukin 6 (IL-6), IL-1 (especially IL-1β), granulocyte-macrophage colony-stimulating factor (GM-CSF), granulocyte colony stimulating factor (G-CSF), and transforming growth factor β1 (TGF-β1).^1,2,18^

 TGF-β which is a multifunctional regulator of cell growth, differentiation, migration and apoptosis has three isoforms. One of these isoforms (TGF-β1) is a potent inducer of metastasis.^18^ Previous studies have shown that elevated levels of inflammatory mediators such as TNF-α and IL-6 are involved in the progression and relapse of breast and head and neck cancers, while inhibition of IL- 6 signaling has been reported to reduce cancer recurrence in preclinical models of breast, head and neck and hepatocellular carcinoma.^1,18^

 VEGF-A, which is a member of both the PDGF and VEGF family of growth factors, stimulates the differentiation and proliferation of endothelial vascular cells and plays an essential role in both physiologic and pathologic angiogenesis. Recent studies have shown that the VEGF-A level is elevated in patients with COVID-19.^19^

 Serum levels of IFN-γ, which is a multifunctional inflammatory cytokine, increase in patients with severe COVID-19. These elevated serum levels could enhance IL-6 production in monocytes.^3^

 IL-6 plays a crucial role in the pathophysiology of cytokine release syndrome. The inflammation caused by its excessive synthesis can induce tissue damage.^20^ By inhibition of apoptosis and stimulation of angiogenesis, it can promote tumor progression and metastasis.^18^ Furthermore, it has been demonstrated that IL-6 plays a significant role in the modulation of cancer-mediated bone destruction.^18^ Apart from its important role in immune-mediated inflammation, IL-6 exerts its effect on tumorigenesis by direct stimulation of cancer cells (promotion of stem cell characteristics, induction of mesenchymal traits and resistance to therapies), as well as indirect action on the TME by making it suitable for metastatic progression of tumoral cells. It also stimulates immune evasion by stabilization of programmed death-ligand 1 (PD-L1).^1,20,21^ Excessive release of IL-6 and other pro-inflammatory cytokines during severe COVID-19 results in extensive activation of NF-κB in both immune and non-immune cells. NF-κB is a protein complex that has several roles in cell survival including controlling the transcription of DNA and production of cytokines.

 Activation of NF-κB can result in reawakening of DCCs in two ways: direct stimulation of cancer cell proliferation, and indirect induction of formation of a pro-metastatic microenvironment.^6^ Apart from pro-tumorigenic effects, IL-6 has also been demonstrated to exert anti-tumor effects by increasing trafficking and adhesiveness of T-cells to the tumor endothelium.^1,22^

 Altogether, it seems that IL-6 signaling is an extremely complex phenomenon with multiple and probably contrasting effects on the TME. The nature of its complicated interactions with tumor cells in patients with COVID-19 needs to be further clarified by future studies. In addition to IL-6, there are reports showing that compared to controls, IL-1b levels are also elevated in patients with COVID-19. IL-1b is responsible for neutrophilic inflammation which enhances the metastatic progression of some types of malignancies such as breast cancer.^23^

 However, similar to IL-6, IL-1b plays dual (and contradictory) roles in the TME: on the one hand, it enhances angiogenesis and metastasis by inducing chronic inflammation and recruitment of myeloid-derived suppressor cells, and on the other, it exerts some anti-tumor effects.^1,24,25^ Therefore, IL-1b also plays a complex role in inflammation and cancer.

 G-CSF and GM-CSF are powerful regulators of proliferation, differentiation and maturation of hematopoietic progenitors and besides, they enhance the effector functions of mature neutrophils.^18^

## Neutrophils and Neutrophil Extracellular Traps

 Until recently, neutrophils were believed to be only involved in defense against extracellular pathogens. However, it is now clear that neutrophils are multi-functional cells whose malfunction or dysregulation can contribute to tumorigenesis.^26^

 Neutrophils and neutrophil extracellular traps (NETs) that are emerging as important players in COVID-19 pathogenesis, are also being recognized as major contributors to DCC reawakening.^1,6-8,27^

 NETs are web-like structures of proteins and DNA. Their main role is to protect the body against infection, particularly by large pathogens, but they are also involved in various immune-mediated conditions.^6,28^

 In addition to entrapment of pathogens and providing a high local concentration of antimicrobial components, NETs are involved in creating a physical barrier that inhibits local access to immune cells and as a result, they contribute to tumor immune evasion and protect tumoral cells from CD8 + T cell- and NK-mediated cytotoxicity.^1,6^

 It has been reported that both tumoral cells and TME control neutrophil recruitment; and high intratumoral density of neutrophils (tumor associated neutrophils, TANs) can regulate tumor progression.^27^ Similar to cytokines, TANs may also exhibit either an antitumor activity (N1 neutrophils), or a pro-tumor activity (N2 neutrophils). While N1 neutrophils are active against tumor growing and metastasis, N2 TANs promote tumor growth, angiogenesis and metastasis.^27^

 The exact mechanism of action of this dual role of TANs in inhibiting or promoting metastatic dissemination of cancer cells is not fully understood. In fact, neutrophils along with their antimicrobial products may contribute to COVID-19-associated inflammation and immunothrombosis, as well as to the reawakening of DCCs, and collectively, to the promotion of tumorigenesis, angiogenesis and metastasis.^1,26,27^

 Neutrophils can induce lymphocyte apoptosis and immune suppression. Once neutrophils reach the TME, they may undergo transition to myeloid-derived suppressor cells (MDSCs) that inhibit CD4 + and CD8 + tumor-infiltrating lymphocytes. Furthermore, MDSCs can stimulate tumor growth and angiogenesis; leading to metastasis development and progression.^1^

 In fact, not only could neutrophils initiate carcinogenesis, but their toxic molecules could also promote the proliferation and spread of tumors.^29^

 It is now revealed that many types of human tumoral cells can produce neutrophil-recruiting chemokines and cytokines that make them able to recruit neutrophils to disable anti-tumor host defenses in the TME.^29^

 A high intratumoral neutrophil density is associated with higher rate of lymph node involvement, higher tumor grade and higher tumor stage.^30^

## Impaired T-Cell Responses

 Lymphopenia and functional impairment of NK cells occur frequently in COVID-19, especially in severe cases. Both CD8 + T-cells and NK cells have a pivotal role in maintaining immune-mediated dormancy and their depletion has been demonstrated to promote DCC reawakening leading to metastatic outgrowth. Therefore, lymphopenia along with inflammation-related factors, may create a microenvironment favorable to metastasis development and progression.^1^

## Can COVID-19 Cure Cancer?

 Contrary to the information provided in this manuscript, there are reports that COVID-19 may have caused cancer remission. Sollini and colleagues reported a case of follicular lymphoma which went into complete remission following SARS-CoV-2 infection.^31^ They suggested that the virus may have firstly induced a local flare phenomenon (as the size of the para-aortic lesion increased initially) and then, exerted its curative abscopal effect. Challenor and Tucker reported a case of Hodgkin lymphoma with progressive lymphadenopathy who went into near complete remission four months after COVID-19.^32^

 They hypothesized that inflammatory cytokines that were released in response to COVID-19 had probably triggered an anti-tumor immune response. This immune response had possibly occurred due to cross-reactivity of pathogen-specific T-cells with tumor antigens and activation of NK cells.

 One explanation could be the fact that spontaneous remission is well-described in various tumors including lymphomas.^33^

 In conclusion, the interaction between COVID-19 and cancer is complex. While many experts have suggested a higher chance of disease recurrence in cancer patients who develop COVID-19, there are reports and proposed mechanisms that argue against this. Further research is needed to improve our understanding of the biology of SARS-CoV-2 virus, and its correlation with cancer prognosis.
